# Patterns of patients with multiple chronic conditions in primary care: A cross-sectional study

**DOI:** 10.1371/journal.pone.0238353

**Published:** 2020-08-31

**Authors:** Xiao Wei Tan, Ying Xie, Jeremy Kaiwei Lew, Poay Sian Sabrina Lee, Eng Sing Lee

**Affiliations:** 1 Institute of Mental Health, Singapore; 2 Clinical Research Unit, National Healthcare Group Polyclinics, Singapore; Universidade Federal de Pelotas, BRAZIL

## Abstract

**Objective:**

Our aim was to identify the patterns of multimorbidity among a group of patients who visited primary care in Singapore.

**Methods:**

A cross-sectional study of electronic medical records was conducted on 437,849 individuals aged 0–99 years who visited National Healthcare Group Polyclinics from 1 Jul 2015 to 30 Jun 2016 for the management of chronic conditions. Patients’ health conditions were coded with the 10^th^ revision of the International Statistical Classification of Diseases and Related Health Problems (ICD-10), and patient records were extracted for analysis. Patients’ diagnosis codes were grouped by exploratory factor analysis (EFA), and patterns of multimorbidity were then identified by latent class analysis (LCA).

**Results:**

EFA identified 19 groups of chronic conditions. Patients with at least three chronic conditions were further separated into eight classes based on demographics and probabilities of various diagnoses. We found that older patients had higher probabilities of comorbid hypertension, kidney disease and ischaemic heart disease (IHD), while younger patients had a higher probability of comorbid obesity. Female patients had higher probabilities of comorbid arthritis and anaemia, while male patients had higher probabilities of comorbid kidney diseases and IHD. Indian patients presented with a higher probability of comorbid diabetes than Chinese and Malay patients.

**Conclusions:**

This study demonstrated that patients with multimorbidity in primary care could be classified into eight patterns. This knowledge could be useful for more precise management of these patients in the multiethnic Asian population of Singapore. Programmes for early intervention for at-risk groups can be developed based on the findings.

## Introduction

Multimorbidity, which is defined as the co-occurrence of multiple chronic conditions in one person [1], is common in primary care [2]. The prevalence of multimorbidity is increasing due to improvements in healthcare services and the resulting longer life expectancies [3–5]. Although it is consistently reported in the literature that the prevalence of multimorbidity increases with age, the majority of the population with multimorbidity is younger than 65 years old [2, 6]. Individuals with multimorbidity have a higher mortality rate [7], a higher risk of inpatient admission [5], and are likely to have a poorer quality of life [7, 8] and reduced physical function than patients who have a single chronic illness [9]. Thus, multimorbidity constitutes a challenge to healthcare services, especially for family physicians in primary care settings [10].

The tendency of some chronic conditions to form clusters has been demonstrated in primary care [11–16]. It has also been reported that the patterns of multimorbidity vary among different age groups, sexes and ethnic groups [17–19]. The identification of the patterns of multimorbidity at earlier stages of one’s life course is important for the prevention and reduction of disease burden in later life. Moreover, health behaviour interventions for chronic conditions, such as improvements in physical activity, are likely to be more effective in the early stages of the disease process [20, 21].

Singapore is a multiethnic urban population with one of the fastest ageing populations worldwide [22]. The prevalence of multimorbidity in both the primary care [23] and general population [24] has been studied and reported. However, a detailed study of the patterns of multimorbidity in the primary care population is lacking. The aim of this study was to use exploratory factor analysis (EFA) to group the diagnoses of interest followed by latent class analysis (LCA) with clinical inputs to identify the patterns of multimorbidity in a large primary care population in Singapore.

## Methods

### Design, settings and study population

A cross-sectional study was conducted on 437,849 patients aged 0–99 years who visited the National Healthcare Group Polyclinics (NHGP) at least once between 1 Jul 2015 and 30 Jun 2016 (both dates inclusive) and who had at least one chronic condition recorded in their clinical records. Patients’ chronic conditions, diagnosed with the 10^th^ revision of the International Statistical Classification of Diseases and Related Health Problems (ICD-10) diagnosis codes [25], were obtained from the electronic medical records. Other information, such as age, sex and ethnicity, was also retrieved. The dataset received by the research team was fully de-identified. Ethics approval was obtained from the National Healthcare Group Domain Specific Review Board (DSRB Reference no: 2018/00466) with waiver of patient consent.

### Statistical analysis

We used descriptive statistics to report patients’ demographic characteristics and chronic conditions. Continuous variables are presented as the mean ± the standard deviation (SD), and categorical variables are presented as the count (percentage).

A total of 93 ICD-10 diagnosis codes were used for the coding of chronic conditions commonly seen in the primary care population at NHGP. We considered a chronic condition to be of significant burden in primary care if its prevalence was at least 0.1%. Twenty-one ICD-10 diagnosis codes had a prevalence of less than 0.1%. Therefore, only 72 ICD-10 diagnosis codes were included for EFA. Each chronic condition was dichotomized into two modalities (present/absent). The correlation matrix between the diagnoses was computed with tetrachoric correlations, and an oblique rotation (Geomin) was performed to confirm the factor structures. We initially screened the eigenvalue of solutions from 2 to 30 factors with all 72 diagnoses. The ideal number was ≤ 22 factors to achieve an eigenvalue ≥ 1. However, convergence could not be attained for some factor solutions, and chi-squared tests could not be computed for some other factor solutions. Convergence could be attained for the 18-factor solution while containing the maximum number of factors.

We next proceeded to remove variables with low-factor loadings (< 0.3) from the 18-factor solution. As we wanted to maximize the number of diagnoses retained, the optimal number of extracted factors for the final solution was determined by keeping the maximum number of factors while meeting the following rules set by the research team: The chi-squared test of model fit needed to be non-significant, root mean square error of approximation (RMSEA) < 0.05, comparative fit index (CFI) > 0.9, Tucker-Lewis index (TLI) > 0.9, standardized root mean square residual (SRMR) < 0.08, factor loading for every included diagnosis ≥ 0.3, and the factors had to be clinically interpretable. With the statistical set of criteria, a final 18-factor solution was obtained. We then considered the clinical relevance and significance of these factors and kept obesity as a standalone factor, despite its factor loading of 0.29, giving a total of 19 factors. As a rule of thumb, factor loadings ≥ 0.30 in absolute value were considered to be significant [26], and the clinical significance of each combination was evaluated before the corresponding diagnoses were selected and grouped as one indicator for LCA.

The LCA was next conducted on the subgroup of patients with multimorbidity. Multimorbidity was defined as having three or more chronic conditions [11, 27]. Fortin et al. reported that defining multimorbidity with a cut-off of three or more chronic conditions could better identify patients with higher care needs in primary care [28]. Patient age was dichotomized into “young (0 to 65 years old)” and “old (65 years old and above)”. Patient demographics (including age, sex and ethnicity) and chronic health conditions derived from EFA were included as indicators for LCA. The initial number of classes that we tested was “2”, and further tests were discontinued when any one of the following criteria was violated: when the *Lo-Mendell-Rubin* adjusted likelihood ratio test (LRT) produced a *p* value < 0.05 [29] when the number of patients in each class was ≥ 5% of the total sample, or when the best log likelihood value could be replicated. In the event that the best log likelihood value could not be replicated due to a local maximum with a default random starting value of (100 10), the random starting number of iterations and the final stage of the optimization to generate more sets of starting values were increased until a replicable solution was found. Several plausible models were compared, and the model that had the lowest adjusted Bayesian information criterion (BIC) value with a *p* value of adjusted LRT < 0.05 was selected as the model of best fit. We stipulated *a priori* that the classification should be clinically meaningful and interpretable. As there were no missing values in our dataset, LCA was performed directly without the need for missing value imputation. To interpret the patterns of multimorbidity, participants with maximum likelihood probabilities of diagnoses ≥ 0.5 were arbitrarily defined as having “high probabilities”. Participants with probabilities of diagnoses of 0.3–0.5 were defined as having “moderate probabilities”, and those with probabilities of diagnoses < 0.3 were defined as having “low probabilities”. Both EFA and LCA were conducted with Mplus (*Muthen and Muthen*, *V8*.*2*).

## Results

A total of 787,446 patient records were extracted from the NHGP electronic medical records system, and a list of diagnoses was drawn up (S1 Table). Among them, 437,849 (55.6%) patients had at least one chronic condition diagnosed, and their data were extracted for EFA to refine the number of diagnoses. The average age of the EFA study cohort was 56 ± 20 years old, 52.3% were female and the majority of the patients were Chinese (73.2%) (Table 1A).

**Table 1:** Patients’ demographics and diagnosis of chronic conditions (A: Total patients; B: Patients with multimorbidity).

A: Total patients (n = 437,849)				B: Patients with multimorbidity (n = 201,348)			
Age (years)		56±20		Age (years)		66±12	
Gender	Female	229,129	52.3%	Gender	Female	104133	51.7%
	Male	208,721	47.7%		Male	97215	48.3%
Ethnicity	Chinese	320,568	73.2%	Ethnicity	Chinese	150584	74.8%
	Malay	58,894	13.5%		Malay	24941	12.4%
	Indian	40,811	9.3%		Indian	19382	9.6%
	Others	17,577	4.0%		Others	6441	3.2%
				Diagnosis	Hyperlipidemia	186443	92.6%
					Hypertension	173479	86.2%
					Diabetes	110724	55.0%
					Arthritis	59304	29.5%
					Kidney disease or failure	46320	23.0%
					Obesity	42359	21.0%
					IHD	36080	17.9%
					Pre-diabetes	33684	16.7%
					Anemia	23801	11.8%
					Liver or gallbladder disease	21867	10.9%
					Stroke or TIA	21963	10.9%
					Heart disease	19143	9.5%
					Thyroid disorder	16922	8.4%
					Asthma or COPD	15421	7.7%
					Depression or anxiety	12435	6.2%
					Dementia or osteoporosis	8941	4.4%
					Poor circulation of lower limbs	6108	3.0%
					Schizophrenia or psychosis	5154	2.6%
					Peripheral vascular disease	4082	2.0%

Abbreviations: COPD: chronic obstructive pulmonary disease; IHD, ischemic heart disease; TIA: transient ischemic attack.

After removing all ICD-10 codes with a prevalence < 0.1%, EFA selected and grouped the remaining diagnosis codes (total 72) into 19 factors. Table 2 summarizes the characteristics and the items included within each factor. The 19 factors included 17 physical conditions and two groups of psychiatric conditions. These factors were hyperlipidaemia, hypertension, diabetes, arthritis, kidney disease or failure, obesity, ischaemic heart disease (IHD), pre-diabetes, anaemia, liver or gallbladder disease, stroke or transient ischaemic attack (TIA), heart diseases, thyroid disorder, asthma or chronic obstructive pulmonary disease (COPD), dementia or osteoporosis, poor circulation of lower limbs, peripheral vascular disease, depression or anxiety, and schizophrenia or psychosis. The factor loadings of each diagnosis are provided in S2 Table. The fit statistics of other solutions by EFA are provided in S3 Table.

**Table 2:** Summary of the grouped diagnoses by exploratory factor analysis.

Original diagnosis	Diagnosis code	Grouped diagnosis
Hyperlipidemia, unspecified	E785	Hyperlipidemia
Essential (primary) hypertension	I10	Hypertension
Type 2 diabetes mellitus without complication	E119	Diabetes
Unspecified diabetes mellitus with background retinopathy	E1431	
Unspecified diabetes mellitus with foot ulcer due to multiple causes	E1473	
Osteoarthritis (OA)–generalized	M159	Arthritis
Arthritis, Unspecified, site unspecified	M1999	
Gout, unspecified, site unspecified	M1099	Kidney disease or failure
Unspecified nephritic syndrome, unspecific	N039	
Chronic kidney disease, unspecified	N189	
Disorder of kidney and ureter, unspecified	N289	
Obesity, unspecified	E669	Obesity
Chronic ischemic heart disease, unspecified	I259	IHD
Congestive heart failure	I500	
Impaired glucose regulation	E09	Pre-diabetes
Impaired glucose regulation without complication	E099	
Thalassemia, unspecified	D569	Anemia
Anemia, unspecified	D649	
Nutritional deficiency, unspecified	E639	
Mental and behavioral disorders due to use of alcohol, acute intoxication	F100	Liver or gallbladder disease
Liver disease, unspecified	K769	
Disease of gallbladder, unspecified	K829	
Carrier of viral hepatitis B	Z2251	
Transient cerebral ischemic attack, unspecified	G459	Stroke or TIA
Stroke, not specified as hemorrhage or infarction	I64	
Atrial fibrillation and flutter	I48	Heart disease
Heart disease, unspecified	I519	
Congenital malformation of heart, unspecified	Q249	
Hypothyroidism, unspecified	E039	Thyroid disorder
Thyrotoxicosis, unspecified	E059	
Endocrine disorder, unspecified	E349	
Chronic obstructive pulmonary disease, unspecified	J449	Asthma or COPD
Asthma, unspecified	J459	
Severe depressive episode without psychotic symptoms, not specified as arising in the postnatal period	F3220	Depression or anxiety
Depressive episode, unspecified, not specified as arising in the postnatal period	F3290	
Anxiety disorder, (unspecified)	F411	
Disorders of initiating and maintaining sleep [insomnias]	G470	
Unspecified dementia	F03	Dementia or osteoporosis
Other osteoporosis, site unspecified	M8199	
Unspecified disorder of bone density and structure, site unspecified	M8599	
Embolism and thrombosis of unspecified vein	I829	Poor circulation of lower limbs
Varicose veins of lower extremities without ulcer or inflammation	I839	
Ulcer of lower limb, not elsewhere classified	L97	
Schizophrenia, unspecified	F209	Schizophrenia or psychosis
Unspecified non-organic psychosis	F29	
Unspecified mental retardation without mention of impairment of behavior	F799	
Epilepsy, unspecified, without mention of intractable epilepsy	G4090	
Neurotic disorder, unspecified	F489	
Atherosclerosis of arteries of extremities, unspecified	I7020	Peripheral vascular disease
Peripheral vascular disease, unspecified	I739	
Other and unspecified disorders of circulatory system	I99	

Abbreviations: COPD: chronic obstructive pulmonary disease; IHD, ischemic heart disease; TIA: transient ischemic attack.

There was a total of 201,348 patients with at least three chronic conditions after being grouped with diagnosis codes generated by EFA. Table 1B shows the demographic distribution of the subgroup sample. The average age of patients with multimorbidity was 66 ± 12 years, and 51.7% of them were female. The prevalence of hyperlipidaemia, hypertension and diabetes was 92.6%, 86.2%, and 55.0%, respectively.

Patients with multimorbidity were divided into eight classes according to the probabilities of diagnosis and demographic variables with LCA (Table 3). Patients in Class 1 were characterized as having high probabilities of hyperlipidaemia (0.92), hypertension (0.96), and diabetes (0.65) and moderate probabilities of kidney disease (0.44), IHD (0.32) and anaemia (0.39). Patients in Class 2 were characterized as having high probabilities of hyperlipidaemia (0.95), hypertension (0.93), and diabetes (0.50) and moderate probabilities of IHD (0.31). Patients in Class 3 had high probabilities of having hyperlipidaemia (0.95), hypertension (0.82), and diabetes (0.64) and moderate probabilities of obesity (0.37). Patients in Class 4 were characterized as having high probabilities of hyperlipidaemia (0.98), hypertension (0.96), and diabetes (0.51) and moderate probabilities of arthritis (0.41). Patients in Class 5 were characterized as having high probabilities of hyperlipidaemia (0.73) and hypertension (0.56) and moderate probabilities of arthritis (0.55). Patients in Class 6 were characterized as having high probabilities of hyperlipidaemia (0.95), hypertension (0.94), and diabetes (0.68) and moderate probabilities of kidney disease (0.42) and IHD (0.31). Patients in Class 7 were characterized as having high probabilities of hyperlipidaemia (0.90), hypertension (0.77), diabetes (0.58), and obesity (0.56) and moderate probabilities of arthritis (0.33). Somewhat similar to Class 7, patients in Class 8 were characterized as having high probabilities of hyperlipidaemia (0.92), hypertension (0.78), and diabetes (0.69) and moderate probabilities of arthritis (0.35) and obesity (0.31).

**Table 3:** Probabilities of patients’ grouped diagnosis within each latent class.

Grouped diagnosis	Class 1 (n = 19564)		Class 2 (n = 49036)		Class 3 (n = 36468)		Class 4 (n = 35778)		Class 5 (n = 13254)		Class 6 (n = 13539)		Class 7 (n = 14189)		Class 8 (n = 19520)	
	probability	p value	probability	p value	probability	p value	probability	p value	probability	p value	probability	p value	probability	p value	probability	p value
Hyperlipidemia	0.92	0.000	0.95	0.000	0.95	0.000	0.98	0.000	0.73	0.000	0.95	0.000	0.90	0.000	0.92	0.000
Hypertension	0.96	0.000	0.93	0.000	0.82	0.000	0.96	0.000	0.56	0.000	0.94	0.000	0.77	0.000	0.78	0.000
Diabetes	0.65	0.000	0.50	0.000	0.64	0.000	0.51	0.000	0.12	0.000	0.68	0.000	0.58	0.000	0.69	0.000
Arthritis	0.20	0.000	0.21	0.000	0.22	0.000	0.41	0.000	0.55	0.000	0.22	0.000	0.33	0.000	0.35	0.000
Kidney disease or failure	0.44	0.000	0.29	0.000	0.14	0.000	0.12	0.000	0.09	0.000	0.42	0.000	0.16	0.000	0.18	0.000
Obesity	0.08	0.000	0.10	0.000	0.37	0.000	0.15	0.000	0.08	0.000	0.25	0.000	0.56	0.000	0.31	0.000
IHD	0.32	0.000	0.31	0.000	0.04	0.000	0.10	0.000	0.04	0.000	0.31	0.000	0.04	0.000	0.22	0.000
Pre-diabetes	0.05	0.000	0.19	0.000	0.21	0.000	0.21	0.000	0.20	0.000	0.09	0.000	0.21	0.000	0.14	0.000
Anemia	0.39	0.000	0.03	0.000	0.04	0.000	0.04	0.000	0.18	0.000	0.17	0.000	0.06	0.000	0.16	0.000
Liver or gallbladder disease	0.05	0.000	0.10	0.000	0.23	0.000	0.07	0.000	0.15	0.000	0.04	0.000	0.12	0.000	0.08	0.000
Stroke or TIA	0.23	0.000	0.14	0.000	0.04	0.000	0.09	0.000	0.07	0.000	0.14	0.000	0.03	0.000	0.08	0.000
Thyroid disorder	0.10	0.000	0.02	0.000	0.07	0.000	0.08	0.000	0.26	0.000	0.05	0.000	0.06	0.000	0.11	0.000
Asthma or COPD	0.07	0.000	0.07	0.000	0.04	0.000	0.05	0.000	0.13	0.000	0.09	0.000	0.14	0.000	0.14	0.000
Depression or anxiety	0.06	0.000	0.03	0.000	0.06	0.000	0.05	0.000	0.21	0.000	0.02	0.000	0.05	0.000	0.06	0.000
Dementia or osteoporosis	0.11	0.000	0.00	0.574	0.00	1.000	0.09	0.000	0.12	0.000	0.03	0.000	0.00	0.012	0.02	0.000
Schizophrenia or psychosis	0.03	0.000	0.01	0.000	0.04	0.000	0.01	0.000	0.06	0.000	0.01	0.000	0.02	0.000	0.03	0.000
Poor circulation of lower limbs	0.05	0.000	0.01	0.000	0.02	0.000	0.03	0.000	0.05	0.000	0.04	0.000	0.03	0.000	0.06	0.000
Peripheral vascular disease	0.06	0.000	0.02	0.000	0.01	0.000	0.01	0.000	0.01	0.000	0.03	0.000	0.01	0.000	0.03	0.000
Heart disease	0.17	0.000	0.11	0.000	0.04	0.000	0.09	0.000	0.12	0.000	0.12	0.000	0.04	0.000	0.07	0.000

Abbreviations: COPD: chronic obstructive pulmonary disease; IHD, ischemic heart disease; TIA: transient ischemic attack.

The eight classes that resulted from LCA were distinctive with regard to age, sex and ethnicity (Table 4). Patients in Class 1 were characterized as having high probabilities of being older (0.95) and Chinese (0.98). All patients in Class 2 were male (1.00) and had a high probability of being Chinese (0.98). Patients in Class 3 were characterized as having high probabilities of being younger (0.91) and Chinese (0.97). Patients in Class 4 were characterized as having high probabilities of being older (0.85), female (0.97) and Chinese (0.99). Patients in Class 5 were characterized as having high probabilities of being female (0.74) and Chinese (0.97). Patients in Class 6 were characterized as having high probabilities of being Malay (0.93). Patients in Class 7 were characterized as having high probabilities of being younger (0.86) and Malay (0.88). Last, patients in Class 8 were characterized as having a high probability of being Indian (0.97).

**Table 4:** Probabilities of patients’ demographics within each latent class.

Demographics	Class 1 (n = 19,564)		Class 2 (n = 49,036)		Class 3 (n = 36,468)		Class 4 (n = 35,778)		Class 5 (n = 13,254)		Class 6 (n = 13,539)		Class 7 (n = 14,189)		Class 8 (n = 19,520)	
	probability	p value	probability	p value	probability	p value	probability	p value	probability	p value	probability	p value	probability	p value	probability	p value
Age <65 years	0.05	0.000	0.40	0.000	0.91	0.000	0.15	0.000	0.56	0.000	0.40	0.000	0.86	0.000	0.60	0.000
Age ≥65 years	0.95	0.000	0.60	0.000	0.09	0.000	0.85	0.000	0.44	0.000	0.60	0.000	0.14	0.000	0.40	0.000
Male	0.43	0.000	1.00	1.000	0.47	0.000	0.03	0.003	0.26	0.000	0.54	0.000	0.34	0.000	0.46	0.000
Female	0.57	0.000	0.00	1.000	0.53	0.000	0.97	0.000	0.74	0.000	0.46	0.000	0.66	0.000	0.54	0.000
Chinese	0.98	0.000	0.98	0.000	0.97	0.000	0.99	0.000	0.97	0.000	0.00	1.000	0.00	1.000	0.00	1.000
Malay	0.00	1.000	0.00	1.000	0.00	1.000	0.00	1.000	0.00	1.000	0.93	0.000	0.88	0.000	0.00	1.000
Indian	0.00	1.000	0.00	1.000	0.00	1.000	0.00	1.000	0.00	1.000	0.00	1.000	0.00	1.000	0.97	0.000

After the combination of the characterization of diagnosis and demographic variables, the resulting groups of patients can be summarized as follows (Table 5): Pattern 1—Older Chinese patients with cardiometabolic diseases, IHD, kidney disease and anaemia; Pattern 2—Chinese male patients with cardiometabolic diseases and IHD; Pattern 3—Younger Chinese patients with cardiometabolic diseases and obesity (Class 3); Pattern 4—Older Chinese female patients with cardiometabolic diseases and arthritis; Pattern 5—Chinese female patients with cardiometabolic diseases and arthritis; Pattern 6—Malay patients with cardiometabolic diseases, IHD and kidney disease; Pattern 7—Younger Malay patients with cardiometabolic diseases, obesity and arthritis; and last, Pattern 8—Indian patients with cardiometabolic diseases, obesity and arthritis.

**Table 5:** Summary of the patterns of multimorbidity by grouped diagnosis of chronic conditions and demographics.

Class	Characteristics
Class 1	Older Chinese patients with cardiometabolic diseases, IHD, kidney disease and anemia
Class 2	Chinese male patients with cardiometabolic diseases and IHD
Class 3	Younger Chinese patients with cardiometabolic diseases and obesity
Class 4	Older Chinese female patients with cardiometabolic diseases and arthritis
Class 5	Chinese female patients with cardiometabolic diseases and arthritis
Class 6	Malay patients with cardiometabolic diseases, IHD and kidney disease
Class 7	Younger Malay patients with cardiometabolic diseases, obesity and arthritis
Class 8	Indian patients with cardiometabolic diseases, obesity and arthritis

Abbreviations: IHD, ischemic heart disease; Younger: Age <65 years old; Older: Age ≥65 years old.

## Discussion

This study provided a comprehensive overview of the unique patterns of multimorbidity among patients who attended the public primary care setting in Singapore. We measured the strength of correlation among a list of chronic conditions using EFA. Utilizing LCA, we defined eight distinct patterns of patients based on the probabilities of chronic conditions and patients’ demographic variables.

We utilized EFA followed by LCA to analyse the patterns of multimorbidity. EFA is based on correlation where the different diseases are treated as a continuous variable. As such, EFA is useful for analysing the correlation between diseases that have a pathophysiological relationship [14]. LCA is a model-based approach that seeks to identify homogeneous groups within a heterogeneous population by hypothesizing an unobserved categorical variable where each category represents a latent class [30]. Patients in the same class share a common probability distribution among the observed variables (e.g., the same disease and demographic probability profile). This makes LCA useful for identifying disease clusters. However, since these clusters are not related to demographic groups, population segments can be constructed post hoc on the basis of the estimated individual factor scores by adding some extra steps to the analytical decisions [31]. Therefore, we adopted EFA to reduce the number of diagnosis codes from the electronic health records and to identify disease clusters, followed by LCA to identify population segments linked with the identified disease clusters to determine the patterns of multimorbidity.

Instead of arbitrarily selecting and grouping diseases for pattern analysis, as most similar studies to date have done, we used algorithms of EFA to reduce the large number of diagnoses as indicators of pattern analysis in our study. With this approach, we were able to select diagnoses with high prevalence and diagnoses sharing similar underlying biological mechanisms. EFA made it possible to combine the accidental, temporary diagnoses, which may be imprecise, ambiguous or incomplete, with those diagnoses that were clinically verified. For example, the diagnosis of an endocrine disorder, which is rather general, was combined with hypothyroidism and thyrotoxicosis. We also identified some unique associations among diseases from different human biological systems. In the current analysis, “gout” was grouped with “nephritic syndrome” as it could result in uric acid nephropathy. Mental and behavioural disorders due to alcohol abuse were grouped with liver or gallbladder diseases, which share common mechanisms of alcohol-related liver hepatitis or cirrhosis. Dementia and osteoporosis were clustered together, as they are closely related to geriatric syndromes of falls and other degenerative conditions related to ageing, such as frailty and sarcopenia. To the best of our knowledge, this is the first time a statistical method, such as EFA, has been utilized to select and reduce the number of chronic conditions from a long list of clinical diagnosis codes instead of the conventional and somewhat arbitrary method of selection by clinical experience.

Estimations of the prevalence and patterns of multimorbidity differ in the literature due to variations in methods, data sources, study populations and diseases included/categorized, making it difficult to compare our results with those of other studies. Instead of the usual criterion of two or more chronic conditions [32], we used a cut-off of three or more chronic conditions as a definition of multimorbidity in this study, as it better identifies patients with higher care needs in primary care.

In this study, patients with multimorbidity showed a high prevalence of cardiometabolic diseases, including hypertension, hyperlipidaemia and diabetes, which was similar to previous studies looking at the adult population in primary care [11, 12]. Other than hypertensive and metabolic diseases, our patients in primary care were also diagnosed with a high prevalence of IHD, chronic kidney diseases, arthritis and obesity. It is not surprising that IHD and chronic kidney disease frequently co-occur with cardiometabolic diseases, as the dysregulation of blood pressure, cholesterol and blood glucose are often associated with coronary heart diseases and chronic kidney disease [33, 34].

The high prevalence of arthritis and obesity in the primary care population of other countries reported by Fortin et al [35] was also observed in our study population. Older adults with arthritis frequently have comorbid cardiovascular disease (CVD), diabetes or hyperlipidaemia [36]. The comorbidity of obesity and diabetes or hypertension has been previously reported among populations in Western countries, and its complex mechanisms have been explicitly discussed [37]. The recognition of highly prevalent comorbid diseases would facilitate the development of appropriate interventional tools for integrated care of patients with common patterns of multimorbidity in primary care.

We observed a low prevalence of psychiatric illnesses in our sample of patients with multimorbidity. It is likely that psychiatric illnesses, including depression and anxiety disorder, are under-diagnosed in primary care, as previous findings have shown [38, 39]. As health professionals who provide first-line medical services for people with chronic conditions, family physicians in primary care should continue to maintain high vigilance in the diagnosis and treatment of mental conditions.

The patterns of multimorbidity identified in our study varied across different age and sex groups. Age was a major risk factor for cardiovascular diseases and metabolic syndrome [40, 41]. It was not surprising that older patients had a higher probability of multimorbidity from combinations of hypertension, kidney disease and IHD [16]. In addition, we observed that younger patients had a higher probability of comorbid obesity, which was also reflected in the findings of obesity rates by age group in the United States [42]. An unhealthy body weight in children and young adults is likely to persist and progress to obesity later in life, leading to an increased risk of other conditions such as diabetes, heart disease and stroke. Lifestyle adjustments and preventive measures are needed to target this specific subgroup of patients to combat the further growth of obesity rates in the nation.

Considerable differences in patterns of multimorbidity between the sexes were observed. In our study population, female patients had higher probabilities of comorbid arthritis and anaemia, while male patients had higher probabilities of comorbid kidney diseases and IHD. Oestrogen may shape arthritis risk, with apparent connections among the age of puberty, childbearing and the use of hormone replacement therapy [43]. Osteoporosis and inflammation in the joints and other tissues can hamper the production of red blood cells in the bone marrow and subsequently cause anaemia among female patients [44]. Male patients are more likely to progress to kidney failure and comorbid heart diseases once they have been diagnosed with chronic kidney diseases [45]. The mechanism remains unclear for sex disparities in comorbid chronic kidney diseases and CVDs.

Singapore is a multiethnic country comprising people mainly from Chinese (76.2%), Malay (15.0%) and Indian (7.4%) descents [46]. The distribution of ethnicity in our study sample of patients with multimorbidity was similar to that of the general population in Singapore. While people with different ethnicities may live in the same environment and share similar public resources, their social culture and lifestyles differ from each other, which may contribute to the different patterns of multimorbidity observed. Although the probabilities of comorbid hypertension and hyperlipidaemia were largely similar among the various ethnicities, Indian patients presented with higher probabilities of comorbid diabetes than Chinese and Malay patients. This finding is possibly due to the higher proportion of carbohydrates often present in a typical Indian diet [47]. The recognition of these social culture patterns may aid in improving diagnostic vigilance in primary care.

The strengths of the current study were the ability to estimate disease prevalence without the errors inherent to self-reported data obtained from surveys and greater statistical power to detect associations of uncommon conditions due to the omission of data from the healthy population. However, our study was limited by the cross-sectional nature of the study design. Moreover, chronic psychiatric conditions may have been under-diagnosed; therefore, the data from this study may not reflect the true prevalence of psychiatric conditions. Other social factors that may be pertinent to the patterns of multimorbidity, such as work and educational status, living conditions and lifestyle factors (including smoking, drinking and dietary habits), were not considered in this study and should be given attention in future research.

## Conclusions

Using an approach combining EFA and LCA, we identified eight patterns of multimorbidity among patients seeking health care in the public primary care setting in Singapore based on ICD10 diagnosis codes, age, sex and ethnicity. Recognizing the common patterns seen in primary care can help clinicians and policymakers target these common patterns by allocating limited healthcare resources optimally. Future research with a prospective study design is needed to further enhance our knowledge and understanding of the mechanisms and causal relationships related to these common patterns of multimorbidity in the primary care population to develop more effective strategies in the prevention, intervention and treatment of multimorbidity.

## Supporting information

S1 Table The prevalence of diagnosis and corresponding diagnosing code extracted from electronic health records. (DOCX)

S2 Table Factor loadings of grouped diagnosis by exploratory factor analysis. (DOCX)

S3 Table Statistic indices of EFA solutions. (DOCX)
