# Supplementary material for: Patterns of patients with multiple chronic conditions in primary care: A cross-sectional study
Source: PLoS One. 2020 Aug 31;15(8):e0238353. doi: 10.1371/journal.pone.0238353 (PMC7458690; doi:10.1371/journal.pone.0238353)
Supplement: S1 Table — (DOCX) [file pone.0238353.s001.docx]

S1 Table: The prevalence of diagnosis and corresponding diagnosing code extracted from electronic health records

| Diagnosis name | Count | Prevalence | Diagnosis code |
| --- | --- | --- | --- |
| Malignant neoplasm without specification of site | 7940 | 1.0% | C80 |
| Thalassemia, unspecified | 3771 | 0.5% | D56.9 |
| Anemia, unspecified | 28786 | 3.7% | D64.9 |
| Disease of blood and blood-forming organs, unspecified | 3929 | 0.5% | D75.9 |
| Hypothyroidism, unspecified | 14133 | 1.8% | E03.9 |
| Thyrotoxicosis, unspecified | 7873 | 1.0% | E05.9 |
| Impaired glucose regulation | 18864 | 2.4% | E09 |
| Impaired glucose regulation without complication | 26630 | 3.4% | E099 |
| Type 1 diabetes mellitus without complication | 3113 | 0.4% | E10.9 |
| Type 2 diabetes mellitus without complication | 124450 | 15.8% | E11.9 |
| Diabetes mellitus with incipient diabetic nephropathy | 914 | 0.1% | E14.2 |
| Diabetes Mellitus with retinopathy | 83 | 0.0% | E14.3 |
| Unspecified diabetes mellitus with background retinopathy | 6269 | 0.8% | E14.31 |
| Unspecified diabetes mellitus with foot ulcer due to multiple causes | 1729 | 0.2% | E14.73 |
| Endocrine disorder, unspecified | 5599 | 0.7% | E34.9 |
| Nutritional deficiency, unspecified | 2219 | 0.3% | E63.9 |
| Obesity, unspecified | 48893 | 6.2% | E66.9 |
| Hyperlipidemia, unspecified | 257114 | 32.7% | E78.5 |
| Unspecified dementia | 3571 | 0.5% | F03 |
| Mental and behavioral disorders due to use of alcohol, acute intoxication | 878 | 0.1% | F10.0 |
| Schizophrenia, unspecified | 2889 | 0.4% | F20.9 |
| Delusional disorder | 92 | 0.0% | F22.9 |
| Unspecified nonorganic psychosis | 1348 | 0.2% | F29 |
| Bipolar affective disorder, unspecified | 51 | 0.0% | F31.9 |
| Severe depressive episode without psychotic symptoms, not specified as arising in the postnatal period | 1700 | 0.2% | F32.20 |
| Depressive episode, unspecified, not specified as arising in the postnatal period | 8880 | 1.1% | F32.90 |
| Anxiety disorder, Unspecified | 6085 | 0.8% | F41.1 |
| Neurotic disorder, unspecified | 1151 | 0.1% | F48.9 |
| Unspecified harmful use of non-dependence producing substance | 111 | 0.0% | F55.9 |
| Unspecified mental retardation without mention of impairment of behavior | 614 | 0.1% | F79.9 |
| Parkinson's disease | 1900 | 0.2% | G20 |
| Epilepsy, unspecified, without mention of intractable epilepsy | 2734 | 0.3% | G40.90 |
| Migraine, unspecified | 6831 | 0.9% | G43.9 |
| Transient cerebral ischemic attack, unspecified | 5158 | 0.7% | G45.9 |
| Disorders of initiating and maintaining sleep [insomnias] | 10468 | 1.3% | G47.0 |
| Trigeminal neuralgia | 635 | 0.1% | G50.0 |
| Hereditary and idiopathic neuropathy, unspecified | 1269 | 0.2% | G60.9 |
| Cerebral palsy, unspecified | 73 | 0.0% | G80.9 |
| Glaucoma, unspecified | 2255 | 0.3% | H40.9 |
| Chronic mucoid otitis media | 2073 | 0.3% | H65.3 |
| Hearing loss, unspecified | 9690 | 1.2% | H91.9 |
| Essential (primary) hypertension | 221760 | 28.2% | I10 |
| Chronic ischemic heart disease, unspecified | 36401 | 4.6% | I25.9 |
| Atrial fibrillation and flutter | 7241 | 0.9% | I48 |
| Congestive heart failure | 3469 | 0.4% | I50.0 |
| Heart disease, unspecified | 17623 | 2.2% | I51.9 |
| Stroke, not specified as hemorrhage or infarction | 19808 | 2.5% | I64 |
| Atherosclerosis of arteries of extremities, unspecified | 1062 | 0.1% | I70.20 |
| Peripheral vascular disease, unspecified | 2598 | 0.3% | I73.9 |
| Embolism and thrombosis of unspecified vein | 1165 | 0.1% | I82.9 |
| Varicose veins of lower extremities without ulcer or inflammation | 5394 | 0.7% | I83.9 |
| Other and unspecified disorders of circulatory system | 1677 | 0.2% | I99 |
| Allergic rhinitis, unspecified | 41059 | 5.2% | J30.4 |
| Chronic obstructive pulmonary disease, unspecified | 5080 | 0.6% | J44.9 |
| Asthma, unspecified | 28778 | 3.7% | J45.9 |
| Gastro-esophageal reflux disease without esophagitis | 20756 | 2.6% | K21.9 |
| Peptic ulcer, unspecified as acute or chronic, without hemorrhage or perforation | 1621 | 0.2% | K27.9 |
| Irritable bowel syndrome without diarrhea | 1517 | 0.2% | K58.9 |
| Liver disease, unspecified | 18658 | 2.4% | K76.9 |
| Disease of gallbladder, unspecified | 3258 | 0.4% | K82.9 |
| Other atopic dermatitis | 18217 | 2.3% | L20.8 |
| Nonscarring hair loss, unspecified | 2719 | 0.3% | L65.9 |
| Other acne | 9252 | 1.2% | L70.9 |
| Ulcer of lower limb, not elsewhere classified | 2235 | 0.3% | L97 |
| Rheumatoid arthritis, unspecified, site unspecified | 2010 | 0.3% | M06.99 |
| Gout, unspecified, site unspecified | 19642 | 2.5% | M10.99 |
| Osteoarthritis (OA) - generalized | 18378 | 2.3% | M15.9 |
| Arthritis, unspecified, site unspecified | 89804 | 11.4% | M19.99 |
| Other osteoporosis, site unspecified | 7283 | 0.9% | M81.99 |
| Unspecified disorder of bone density and structure, site unspecified | 831 | 0.1% | M85.99 |
| Unspecified nephritic syndrome, unspecified | 770 | 0.1% | N03.9 |
| Chronic kidney disease, unspecified | 21638 | 2.7% | N18.9 |
| Disorder of kidney and ureter, unspecified | 21112 | 2.7% | N28.9 |
| Hyperplasia of prostate | 13031 | 1.7% | N40 |
| Menopausal and perimenopausal disorder, unspecified | 2576 | 0.3% | N95.9 |
| Female infertility, unspecified | 1818 | 0.2% | N97.9 |
| Congenital malformation of eye, unspecified | 255 | 0.0% | Q15.9 |
| Congenital malformation of heart, unspecified | 460 | 0.1% | Q24.9 |
| Congenital malformation of digestive system, unspecified | 14 | 0.0% | Q45.9 |
| Congenital malformation of male genital organ, unspecified | 74 | 0.0% | Q55.9 |
| Congenital malformation of urinary system, unspecified | 119 | 0.0% | Q64.9 |
| Congenital malformation of musculoskeletal system, unspecified | 837 | 0.1% | Q79.9 |
| Congenital malformation, unspecified | 295 | 0.0% | Q89.9 |
| Down's syndrome, unspecified | 121 | 0.0% | Q90.9 |
| Unspecified urinary incontinence | 3417 | 0.4% | R32 |
| Carrier of viral hepatitis B | 8737 | 1.1% | Z22.51 |
| Acquired absence of foot and ankle | 122 | 0.0% | Z89.4 |
| Acquired absence of leg at or below knee | 178 | 0.0% | Z89.5 |
| Acquired absence of leg above knee | 68 | 0.0% | Z89.6 |
