# Supplementary material for: Patterns of patients with multiple chronic conditions in primary care: A cross-sectional study
Source: PLoS One. 2020 Aug 31;15(8):e0238353. doi: 10.1371/journal.pone.0238353 (PMC7458690; doi:10.1371/journal.pone.0238353)
Supplement: S2 Table — (DOCX) [file pone.0238353.s002.docx]

S2 Table: Factor loadings of grouped diagnosis by exploratory factor analysis

| Diagnosis code | Diagnosis name | Factor 1 | Factor 2 | Factor 3 | Factor 4 | Factor 5 | Factor 6 | Factor 7 | Factor 8 | Factor 9 | Factor 10 | Factor 11 | Factor 12 | Factor 13 | Factor 14 | Factor 15 | Factor 16/17 | Factor 18 | Factor 19 |
| --- | --- | --- | --- | --- | --- | --- | --- | --- | --- | --- | --- | --- | --- | --- | --- | --- | --- | --- | --- |
| E785 | Hyperlipidemia, unspecified | 1.39 |  |  |  |  |  |  |  |  |  |  |  |  |  |  |  |  |  |
| I10 | Essential (primary) hypertension |  | 1.84 |  |  |  |  |  |  |  |  |  |  |  |  |  |  |  |  |
| E119 | Type 2 diabetes mellitus without complication |  |  | 1.26 |  |  |  |  |  |  |  |  |  |  |  |  |  |  |  |
| E1431 | Unspecified diabetes mellitus with background retinopathy |  |  | 0.53 |  |  |  |  |  |  |  |  |  |  |  |  |  |  |  |
| E1473 | Unspecified diabetes mellitus with foot ulcer due to multiple causes |  |  | 0.38 |  |  |  |  |  |  |  |  |  |  |  |  |  |  |  |
| M159 | Osteoarthritis (OA) - generalized |  |  |  | 0.54 |  |  |  |  |  |  |  |  |  |  |  |  |  |  |
| M1999 | Arthritis, unspecified, site unspecified |  |  |  | 0.66 |  |  |  |  |  |  |  |  |  |  |  |  |  |  |
| M1099 | Gout, unspecified, site unspecified |  |  |  |  | 0.51 |  |  |  |  |  |  |  |  |  |  |  |  |  |
| N039 | Unspecified nephritic syndrome, unspecified |  |  |  |  | 0.37 |  |  |  |  |  |  |  |  |  |  |  |  |  |
| N189 | Chronic kidney disease, unspecified |  |  |  |  | 0.74 |  |  |  |  |  |  |  |  |  |  |  |  |  |
| N289 | Disorder of kidney and ureter, unspecified |  |  |  |  | 0.47 |  |  |  |  |  |  |  |  |  |  |  |  |  |
| I259 | Chronic ischemic heart disease, unspecified |  |  |  |  |  | 0.47 |  |  |  |  |  |  |  |  |  |  |  |  |
| I500 | Congestive heart failure |  |  |  |  |  | 0.62 |  |  |  |  |  |  |  |  |  |  |  |  |
| E09 | Impaired glucose regulation |  |  |  |  |  |  | 0.79 |  |  |  |  |  |  |  |  |  |  |  |
| E099 | Impaired glucose regulation without complication |  |  |  |  |  |  | 0.78 |  |  |  |  |  |  |  |  |  |  |  |
| D569 | Thalassemia, unspecified |  |  |  |  |  |  |  | 0.54 |  |  |  |  |  |  |  |  |  |  |
| D649 | Anemia, unspecified |  |  |  |  |  |  |  | 0.99 |  |  |  |  |  |  |  |  |  |  |
| E639 | Nutritional deficiency, unspecified |  |  |  |  |  |  |  | 0.34 |  |  |  |  |  |  |  |  |  |  |
| F100 | Mental and behavioural disorders due to use of alcohol, acute intoxication |  |  |  |  |  |  |  |  | 0.38 |  |  |  |  |  |  |  |  |  |
| K769 | Liver disease, unspecified |  |  |  |  |  |  |  |  | 0.70 |  |  |  |  |  |  |  |  |  |
| K829 | Disease of gallbladder, unspecified |  |  |  |  |  |  |  |  | 0.61 |  |  |  |  |  |  |  |  |  |
| Z2251 | Carrier of viral hepatitis B |  |  |  |  |  |  |  |  | 0.56 |  |  |  |  |  |  |  |  |  |
| G459 | Transient cerebral ischaemic attack, unspecified |  |  |  |  |  |  |  |  |  | 0.47 |  |  |  |  |  |  |  |  |
| I64 | Stroke, not specified as haemorrhage or infarction |  |  |  |  |  |  |  |  |  | 0.73 |  |  |  |  |  |  |  |  |
| I48 | Atrial fibrillation and flutter |  |  |  |  |  |  |  |  |  |  | 0.49 |  |  |  |  |  |  |  |
| I519 | Heart disease, unspecified |  |  |  |  |  |  |  |  |  |  | 0.46 |  |  |  |  |  |  |  |
| Q249 | Congenital malformation of heart, unspecified |  |  |  |  |  |  |  |  |  |  | 0.47 |  |  |  |  |  |  |  |
| E039 | Hypothyroidism, unspecified |  |  |  |  |  |  |  |  |  |  |  | 0.59 |  |  |  |  |  |  |
| E059 | Thyrotoxicosis, unspecified |  |  |  |  |  |  |  |  |  |  |  | 0.63 |  |  |  |  |  |  |
| E349 | Endocrine disorder, unspecified |  |  |  |  |  |  |  |  |  |  |  | 0.42 |  |  |  |  |  |  |
| J449 | Chronic obstructive pulmonary disease, unspecified |  |  |  |  |  |  |  |  |  |  |  |  | 0.40 |  |  |  |  |  |
| J459 | Asthma, unspecified |  |  |  |  |  |  |  |  |  |  |  |  | 0.86 |  |  |  |  |  |
| F3220 | Severe depressive episode without psychotic symptoms, not specified as arising in the postnatal period |  |  |  |  |  |  |  |  |  |  |  |  |  | 0.71 |  |  |  |  |
| F3290 | Depressive episode, unspecified, not specified as arising in the postnatal period |  |  |  |  |  |  |  |  |  |  |  |  |  | 0.79 |  |  |  |  |
| F411 | Anxiety disorder, Unspecified |  |  |  |  |  |  |  |  |  |  |  |  |  | 0.60 |  |  |  |  |
| G470 | Disorders of initiating and maintaining sleep [insomnias] |  |  |  |  |  |  |  |  |  |  |  |  |  | 0.50 |  |  |  |  |
| F03 | Unspecified dementia |  |  |  |  |  |  |  |  |  |  |  |  |  |  | 0.42 |  |  |  |
| M8199 | Other osteoporosis, site unspecified |  |  |  |  |  |  |  |  |  |  |  |  |  |  | 0.58 |  |  |  |
| M8599 | Unspecified disorder of bone density and structure, site unspecified |  |  |  |  |  |  |  |  |  |  |  |  |  |  | 0.55 |  |  |  |
| E669 | Obesity, unspecified* |  |  |  |  |  |  |  |  |  |  |  |  |  |  |  | 0.29 |  |  |
| I829 | Embolism and thrombosis of unspecified vein |  |  |  |  |  |  |  |  |  |  |  |  |  |  |  | 0.37 |  |  |
| I839 | Varicose veins of lower extremities without ulcer or inflammation |  |  |  |  |  |  |  |  |  |  |  |  |  |  |  | 0.52 |  |  |
| L97 | Ulcer of lower limb, not elsewhere classified |  |  |  |  |  |  |  |  |  |  |  |  |  |  |  | 0.61 |  |  |
| F209 | Schizophrenia, unspecified |  |  |  |  |  |  |  |  |  |  |  |  |  |  |  |  | 0.91 |  |
| F29 | Unspecified nonorganic psychosis |  |  |  |  |  |  |  |  |  |  |  |  |  |  |  |  | 0.83 |  |
| F799 | Unspecified mental retardation without mention of impairment of behaviour |  |  |  |  |  |  |  |  |  |  |  |  |  |  |  |  | 0.62 |  |
| G4090 | Epilepsy, unspecified, without mention of intractable epilepsy |  |  |  |  |  |  |  |  |  |  |  |  |  |  |  |  | 0.32 |  |
| F489 | Neurotic disorder, unspecified |  |  |  |  |  |  |  |  |  |  |  |  |  |  |  |  | 0.51 |  |
| I7020 | Arthritis, unspecified, site unspecified |  |  |  |  |  |  |  |  |  |  |  |  |  |  |  |  |  | 1.00 |
| I739 | Peripheral vascular disease, unspecified |  |  |  |  |  |  |  |  |  |  |  |  |  |  |  |  |  | 0.91 |
| I99 | Other and unspecified disorders of circulatory system |  |  |  |  |  |  |  |  |  |  |  |  |  |  |  |  |  | 0.36 |

*Obesity was kept as a single factor due to high prevalence
