# Supplementary material for: Patterns of patients with multiple chronic conditions in primary care: A cross-sectional study
Source: PLoS One. 2020 Aug 31;15(8):e0238353. doi: 10.1371/journal.pone.0238353 (PMC7458690; doi:10.1371/journal.pone.0238353)
Supplement: S3 Table — (DOCX) [file pone.0238353.s003.docx]

S3 Table: Statistic indices of EFA solutions

| EFA solutions | Chi-square test p value | CFI | TLI | SRMR |
| --- | --- | --- | --- | --- |
| 2-factors solution | <0.001 | 0.937 | 0.928 | 0.128 |
| 3-factors solution | <0.001 | 0.958 | 0.949 | 0.102 |
| 4-factors solution | <0.001 | 0.966 | 0.957 | 0.084 |
| 5-factors solution | <0.001 | 0.972 | 0.962 | 0.079 |
| 6-factors solution | <0.001 | 0.978 | 0.968 | 0.068 |
| 7-factors solution | <0.001 | 0.984 | 0.975 | 0.061 |
| 8-factors solution | <0.001 | 0.984 | 0.974 | 0.057 |
| 9-factors solution | <0.001 | 0.991 | 0.983 | 0.059 |
| 10-factors solution | <0.001 | 0.987 | 0.978 | 0.054 |
| 11-factors solution | <0.001 | 0.991 | 0.982 | 0.041 |
| 12-factors solution | no converge | | | |
| 13-factors solution | chi-square test cannot be computed | | | |
| 14-factors solution | <0.001 | 0.996 | 0.992 | 0.038 |
| 15-factors solution | <0.001 | 0.997 | 0.994 | 0.027 |
| 16-factors solution | <0.001 | 0.997 | 0.993 | 0.031 |
| 17-factors solution | chi-square test cannot be computed | | | |
| 18-factors solution | <0.001 | 0.998 | 0.996 | 0.030 |
| 19-factors solution | contains factor with all loadings <0.3 | | | |
| 20-factors solution | chi-square test cannot be computed | | | |
| 21-factors solution | chi-square test cannot be computed | | | |
| 22-factors solution | chi-square test cannot be computed | | | |
